# Outcomes of 23-Gauge Vitrectomy Combined with Phacoemulsification, Panretinal Photocoagulation, and Trabeculectomy without Use of Anti-VEGF Agents for Neovascular Glaucoma with Vitreous Hemorrhage

**DOI:** 10.1155/2016/3097379

**Published:** 2016-01-17

**Authors:** Hua Yan

**Affiliations:** Department of Ophthalmology, Tianjin Medical University General Hospital, No. 154, Anshan Road, Tianjin 300052, China

## Abstract

*Purpose*. To evaluate the outcomes of 23-gauge vitrectomy combined with phacoemulsification, PRP and trabeculectomy without use of anti-VEGF-agents for NVG.* Methods*. Eighteen eyes of 18 patients with NVG underwent 23-gauge vitrectomy combined with phacoemulsification, PRP and trabeculectomy without use of anti-VEGF agents. The preoperative BCVA ranged from light perception to 0.2. The preoperative IOP ranged from 38 mmHg to 64 mmHg with a mean of 54 ± 8 mmHg. The average follow-up time was 14.5 ± 3 months with a range from 11 to 24 months.* Results*. The postoperative VA increased in 14 eyes and was stable in 4 eyes at the final follow-up. The mean IOP was 12 ± 3 mmHg at postoperative day 1. The mean IOP was 15 ± 2 mmHg, 16 ± 3 mmHg, 23 ± 5 mmHg, 28 ± 4 mmHg, 22 ± 5 mmHg, 17 ± 3 mmHg, and 19 ± 4 mmHg at postoperative days 2 and 3, 1, 2, 3, and 12 weeks, and 1 year postoperatively, respectively, with a range from 10 to 30 mmHg at the final follow-up time point of one year. The IOP was significantly lower than the preoperative one 12 weeks postoperatively (*p* < 0.05).* Conclusion*. 23-gauge vitrectomy combined with phacoemulsification, PRP, and trabeculectomy without use of anti-VEGF-agents is a safe and effective method in treating NVG.

## 1. Introduction

Neovascular glaucoma (NVG) complicated by vitreous hemorrhage (VH) is commonly caused by retinal ischemia secondary to central retinal vein occlusion (CRVO), branch retinal vein occlusion (BRVO), and/or proliferative diabetic retinopathy (PDR) [1–3]. Vision can be severely affected by NVG, which often causes permanent damage to the optic nerve secondary to high intraocular pressures associated with angle closure. Ischemic neovascular vessels can form and occlude the angle. Hypoxia caused by CRVO, BRVO, and PDR induces production and release of vascular endothelial growth factor (VEGF) and inflammatory mediators. These neovascular vessels are leaky and fragile and can cause VH and NVG and are also associated with leakage of inflammatory molecules which can cause macular edema.

NVG is one of the most recalcitrant glaucoma types to treatment and has one of the worst outcomes of the many types of glaucoma. NVG often needs surgical treatment because medical treatment of elevated IOP is often inadequate. The standard of early treatment once NVG occurs is panretinal photocoagulation (PRP), which destroys ischemic retina and decreases the production of proangiogenic factors such as VEGF. PRP can prevent ischemic retina from progressing to NVG. Glaucoma drainage implants, trabeculectomy with mitomycin C, cyclocryotherapy, or diode laser coagulation of the ciliary body is another treatment option available for the treatment of recalcitrant and end-stage NVG [4–6]. Pars plana vitrectomy (PPV) combined with glaucoma drainage implantation can produce good control of intraocular pressure (IOP) in NVG patients with PDR [7, 8].

Antivascular endothelial growth factor (anti-VEGF) molecules have been used for many ocular diseases, including NVG. A number of studies have evaluated the use of anti-VEGF agents as stand-alone or adjunctive treatment for NVG [9]. Adjunctive anti-VEGF treatment promotes the surgical success rate for NVG. IOP lowering surgery combined with PRP or intravitreal injection of anti-VEGF antibodies aids in regression of neovascularization and stabilization of vision [10, 11].

The optimal approaches to treating NVG with VH are to provide patients with an individualized management plan according to etiology, stage of disease, and visual potential among other factors. PPV combined with PRP can reduce the occurrence of NVG and increases visual acuity (VA) in CRVO with VH [12]. In this retrospective study, we investigated the long term surgical outcomes, including VA and IOP of NVG eyes complicated with VH, after treatment with 23-gauge vitrectomy combined with phacoemulsification, PRP, and trabeculectomy without use of anti-VEGF agents. We suggest that this is an effective method for the treatment of NVG induced by retinal vessel occlusion and PDR.

## 2. Patients and Methods

### 2.1. Patients

The study was approved by Tianjin Medical University General Hospital Medical Ethics Committee and complies with the Declaration of Helsinki, including current revisions, and with the Good Clinical Practice guidelines. The procedures followed were in accordance with institutional guidelines; all the subjects provided their written informed consent for sampling according to the Declaration of Helsinki. All the subjects were recruited from the ophthalmology department of Tianjin General Hospital.

**Figure 1:**
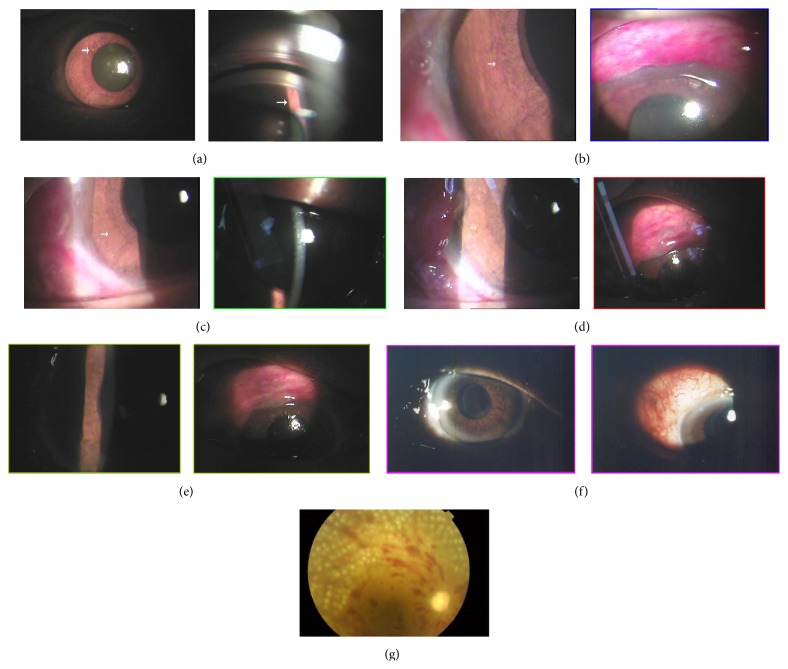
A 63-year-old woman with a history of diabetes mellitus complicated with renal failure underwent combined surgery for PDR complicated with NVG. The preoperative VA was LP and increased to 0.04 postoperatively, with IOP decreasing from 64 mmHg preoperatively to 19 mmHg at the final follow-up postoperatively. Rubeosis iridis was present in the iris preoperatively and regressed 1 week postoperatively. At the final follow-up postoperatively, rubeosis iridis disappeared in the iris and conjunctiva filtering bleb was flat. (a) Rubeosis iridis (white arrow) was present in the iris preoperatively, and new vessels (white arrow) were seen at the anterior chamber angle by gonioscopy. IOP was 64 mmHg. (b) Rubeosis iridis (white arrow) was still present in the iris 1 day postoperatively, and conjunctiva filtering bleb was formed. IOP was 16 mmHg. (c) Rubeosis iridis (white arrow) was significantly decreased in the iris 2 days postoperatively, and cornea was clear. IOP was 15 mmHg. (d) Rubeosis iridis (white arrow) was not significant in the iris 3 days postoperatively, and conjunctiva filtering bleb was obvious. IOP was 15 mmHg. (e) Rubeosis iridis regressed in the iris 1 week postoperatively, and conjunctiva filtering bleb was stable. IOP was 15 mmHg. (f) Rubeosis iridis disappeared in the iris at the final follow-up postoperatively, and conjunctiva filtering bleb was flat. IOP was 19 mmHg. (g) Intraoperative PRP for treatment of PDR complicated with BRVO in patients with NVG and optic nerve atrophy 1 week postoperatively.

Eighteen eyes of 18 consecutive patients who had NVG and VH underwent 23-gauge vitrectomy combined with phacoemulsification, PRP, and trabeculectomy without use of anti-VEGF agents from January 2012 to June 2014. Ten patients were males, and 8 were females. The age ranged from 58 to 76 years with a mean of 62 ± 6 years. Three patients were with hypertension, 7 patients were with diabetes mellitus complicated with hypertension, and 8 patients were with diabetes mellitus complicated with renal failure. Eight eyes had a history of CRVO, 6 eyes had PDR complicated with BRVO, and 4 eyes had PDR alone (Table 1). The rubeosis iridis conditions were present in 18 NVG eyes preoperatively (Figure 1). The preoperative VA ranged from light perception to 0.2. The preoperative IOP ranged from 38 to 64 mmHg with a mean of 54 ± 8 mmHg. The average follow-up was 14.5 ± 3 months with a range from 12 to 24 months.

Eyes with a prior history of intravitreal injection of steroids or anti-VEGF agents, VH without retinal vessels occlusion and PDR, ocular trauma, ocular tumors, and corneal opacity precluding PRP, or cataract extraction before NVG was diagnosed were excluded.

### 2.2. Pre- and Postoperative Examinations

Pre- and postoperative examinations included VA, slit-lamp examination, gonioscopy, indirect ophthalmoscopy, IOP, and B-scan.

### 2.3. Surgical Procedures

We performed 23-gauge PPV using a three-port technique in all patients. The eye received retrobulbar and peribulbar 2% lidocaine for anesthesia, and the eye was then prepared for a standard three-port 23-gauge vitrectomy. After the infusion cannula was placed, the cataract was extracted by phacoemulsification through a clear corneal incision approach. The posterior capsule was preserved in 10 eyes and was completely resected in 8 eyes. During PPV, posterior hyaloid separation was induced by suction using the vitreous cutter over the optic nerve head in eyes without posterior vitreous detachment. In each case, the meticulous shaving of the vitreous base under a wide-angle viewing system with assisted sclera depression was performed to remove as much residual blood as possible. After the VH was completely removed, any fibrovascular tissue present was removed using the microvitrector tip. Hemostasis was maintained by raising the IOP through the infusion fluid or by using endodiathermy intraoperatively.

PRP was performed in all eyes, and subsequent peripheral retinal cryotherapy was given only in severe NVG eyes during the surgery. A 4 × 3 mm lamellar sclera flap was created in the superior region to the limbal border, and then a mitomycin C trabeculectomy was performed. Postoperative examinations were completed at 1, 2, and 3 days, 1, 2, 3, and 12 weeks, and 1 year after the surgery. Intraocular lenses were implanted in 3 eyes 3 months after the combined surgery. No supplemental PRP was given in the postoperative period.

Paired Student's *t*-test was used to analyze changes in pre- and postoperative IOP.

## 3. Results

After the combined surgery, the conjunctival incisions healed well, and the conjunctival flap was formed significantly. Corneas were clear, and iridectomies were patent. Rubeosis iridis has regressed in all eyes 1 week postoperatively (Figure 1). At the final follow-up postoperatively, rubeosis iridis disappeared in the iris and conjunctiva filtering bleb was flat.

### 3.1. VA

**Figure 2:**
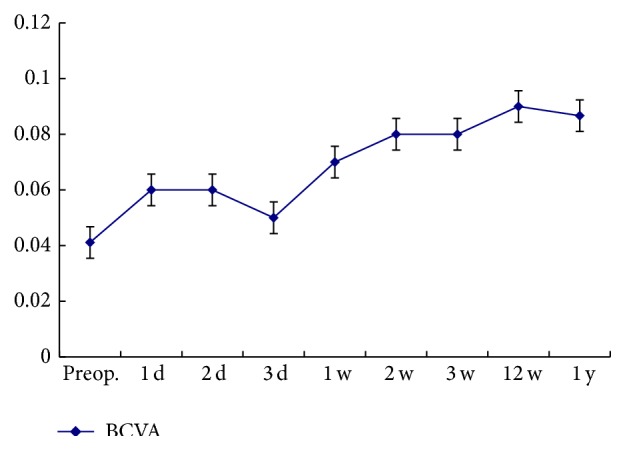
The chart clearly demonstrates the change trend of BCVA pre- and postoperatively. The BCVA significantly increased within three months postoperatively compared with the preoperative BCVA (*p* < 0.05) and then remained stable (*p* > 0.05).

The postoperative VA increased in 14 eyes with the BCVA ranging from 0.02 to 0.4 and was stable relative to the preoperative vision in 4 eyes at the final follow-up. The change trend of BCVA pre- and postoperatively was demonstrated in Figure 2. The BCVA significantly increased within three months postoperatively compared with the preoperative BCVA (*p* < 0.05) and then remained stable (*p* > 0.05).

### 3.2. IOP

**Figure 3:**
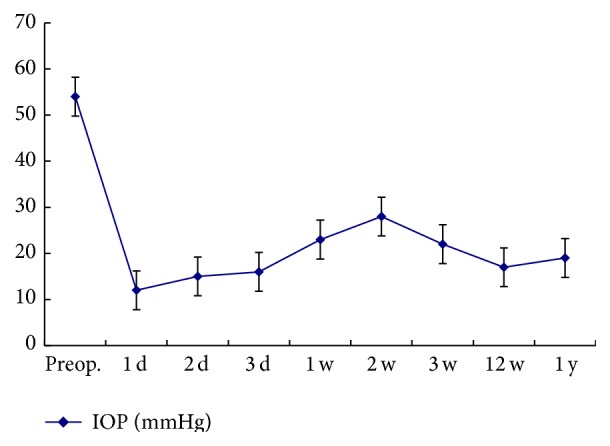
The chart clearly demonstrates the change trend of IOP pre- and postoperatively. The IOP was significantly lower within three months postoperatively compared with the preoperative baseline IOP (*p* < 0.05) and then remained stable (*p* > 0.05).

The mean IOP was 12 ± 3 mmHg, 15 ± 2 mmHg, 16 ± 3 mmHg, 23 ± 5 mmHg, 28 ± 4 mmHg, 22 ± 5 mmHg, 17 ± 3 mmHg, and 19 ± 4 mmHg at 1, 2, and 3 days, 1, 2, 3, and 12 weeks, and 1 year, postoperatively. The IOP ranged from 10 to 30 mmHg 1 year postoperatively. The IOP was significantly lower at three months compared to the preoperative baseline IOP (*p* < 0.05) and then remained stable to the final follow-up (*p* > 0.05) (Figure 3). IOP was not in normal range in 8 eyes 1 month postoperatively, and 2% carteolol hydrochloride was used twice a day for 1 month in 5 eyes and combined with brinzolamide 3 times a day for 1 month in 3 eyes for making IOP to normal range.

### 3.3. Postoperative Complications

Postoperative complications mainly included fibrosis exudates in the anterior chamber (7 eyes), temporary IOP elevation at 2 weeks postoperatively (2 eyes), and postoperative suprachoroidal hemorrhage (1 eye).

## 4. Discussion

A cyclodestructive method or glaucoma drainage surgery is frequently the final option for treatment of severe NVG, but the postoperative VA rarely increases, and IOP is sometimes poorly controlled after aggressive treatment [4, 5]. We treated NVG using 23-gauge vitrectomy combined with phacoemulsification, PRP, and trabeculectomy without use of anti-VEGF agents and obtained predictable results.

NVG develops secondary to extensive ischemic retinal changes, such as occurring with PDR and CRVO. In CRVO, when the retinal vein is occluded, the ischemic retina releases VEGF and inflammatory cytokines into the vitreous cavity, posterior chamber, anterior chamber, and anterior chamber angle [13]. The VEGF in the anterior chamber stimulates neovascularization of the iris and the angle, restricts aqueous outflow, and results eventually in the development of NVG. Wakabayashi et al. [14] reported high intraocular VEGF level at the time of primary vitrectomy in patients with PDR, which was identified as a significant risk factor for postoperative early VH. Goto et al. [15] reported that the risk factors for NVG after vitrectomy in eyes with PDR are independently associated with male sex, younger age, higher baseline IOP, preoperative neovascularization in the angle, and NVG in the fellow's eye.

In this study, 8 patients had hypertension complicated with CRVO, 6 patient had hypertension complicated with BRVO, and 4 patients had PDR. The 4 patients with PDR were also complicated with either CRVO or BRVO. PDR in these 4 patients was not very severe. Therefore, the main reason resulting in NVG was either CRVO or BRVO. In our experience, NVG caused by CRVO or BRVO occurs more quickly than by diabetic retinopathy. In patients with CRVO or BRVO, the observation of IOP, rubeosis irides, and retinal neovascularization should be emphasized, and PRP should be performed for decreasing the risk of developing NVG.

Phacoemulsification, vitrectomy, PRP, and trabeculectomy were adopted to treat NVG complicated with VH according to the different treatment mechanisms. Phacoemulsification to remove a cataract can improve the view so vitrectomy and PRP can be performed more easily. In patients without a posterior capsule preserved after phacoemulsification, the postoperative IOP may be lowered significantly because of the free communication of aqueous humor between the anterior chamber and vitreous cavity. However, postoperative suprachoroidal hemorrhage occasionally occurred because of sudden decrease of postoperative IOP or simultaneously ocular trauma. In this study, 1 eye without posterior capsule preservation developed suprachoroidal hemorrhage because of ocular trauma 3 days postoperatively and were treated with drainage of suprachoroidal hemorrhage through sclerotomies and placement of 30% C3F8 into the vitreous cavity.

The benefits of vitrectomy for NVG complicated by VH are as follows: (1) vitrectomy removes VH and clears the vitreous cavity which can increase VA, (2) vitrectomy removes and may reduce the expression of VEGF, which is a vital factor for neovascularization, (3) vitrectomy to remove VH can prevent hemolytic or ghost cell glaucoma, and (4) PRP can be performed completely and easily after PPV. In NVG with VH, performing complete PRP is often difficult even in cases with minimal VH.

PRP, the standard of care for treating NVG in ischemic CRVO and PDR, decreases oxygen consumption and production of VEGF and aids in the regression of rubeosis irides. In patients with previous partial retinal laser treatment, additional laser photocoagulation can be performed intraoperatively because it is conducted under anesthesia and patient feels no or minimal pain which allows for more complete PRP along with the greater peripheral retinal view for additional PRP. Rubeosis iridis does not regress immediately after PRP, and effect on IOP can be minimal or delayed after PRP. Therefore, in patients with open angle NVG, trabeculectomy is effective in decreasing the IOP temporarily as, in patients with angle closure NVG, trabeculectomy is used to decrease the IOP for a longer term.

In recent years, anti-VEGF agents have been widely applied for the treatment of ischemic retinopathy, including retinal vessel occlusion and PDR. Anti-VEGF agents used in PDR can reduce intra- and postoperative hemorrhage associated with the surgical removal of VH. Although very little information about the role of anti-VEGF treatment in NVG complicated with VH exists, trabeculectomy combined with injection of anti-VEGF agents into the vitreous body for NVG has been documented. However, the long term effect of decreasing the IOP does not appear to be significant compared with trabeculectomy alone. Anti-VEGF agents can cause temporary regression of iris neovascularization, but the long term use for anti-VEGF agents for NVG is not as clear as it is for age-related macular degeneration. Additionally, long term use of anti-VEGF agents for NVG can be very expensive.

Other protocols for treatment of NVG have been described. Bartz-Schmidt et al. [16] treated NVG with vitrectomy combined with PRP, direct laser coagulation of ciliary processes, and silicone oil tamponade. The IOP normalized in 59% of eyes at 6 months postoperatively and in 72% of eyes after 1 year. Chuang et al. [12] reported 56 eyes with CRVO complicated by VH which underwent PPV combined with PRP, and the most important result was a lower incidence of NVG development and VA improvement. Kinoshita et al. [17] reported PPV combined with lensectomy with anterior capsule preservation, endophotocoagulation, and silicon oil tamponade for NVG. The IOP decreased from 29 ± 19 mmHg to 17 ± 6 mmHg 1 year postoperatively with a success rate of 69.2%. Luttrull and Avery [18] reported the treatment of NVG with vitrectomy combined with pars plana glaucoma drainage implant, and all patients had normal IOPs 1 year postoperatively. Wallsh et al. [7] reported pars plana placement of Ahmed valved glaucoma drainage implants in combination with PPV in the treatment of NVG. The IOP decreased from 37.6 mmHg to 13.8 mmHg. Jeong et al. [8] reported pars plana Ahmed GDI placement combined with 23-gauge vitrectomy for NVG in DR, and IOP decreased from 35.9 ± 6.3 mmHg to 13.3 ± 3.2 mmHg at the last visit. Control of IOP was achieved in all patients, but 91% needed antiglaucoma medications. Sevim et al. [19] reported the effect of intravitreal bevacizumab injection before Ahmed glaucoma valve implantation in NVG and the surgical success rate was 79%. In this study, the IOP was normal in 71.4% during postoperative follow-up, and the neovascularization of the iris disappeared in all eyes. Preoperative use of bevacizumab may be to have a better result when our combined surgery is not practical or feasible [20].

In conclusion, vitrectomy combined with phacoemulsification, PRP, and trabeculectomy without use of anti-VEGF agents is a safe and effective method in treating NVG complicated with VH. In some cases, loss of VA was decreased. This combined surgery may be considered as a first treatment option for NVG complicated VH, and the long term effects of the combined surgery should be investigated.

## Figures and Tables

**Table 1:** Clinical data of patients with NVG.

Case number	Sex	Age (y)	Systemic disease	Diagnosis	Previous treatment	BCVA		IOP (mmHg)		Complications	Follow-up (months)
						Preop.	Postop.	Preop.	Postop.		
1	M	76	Hypertension	CRVO	B + C	0.02	0.08	58	21	Fibrosis exudates in AC	16
2	M	61	DM + hypertension	CRVO	B + C, partial laser	0.04	0.04	53	22	None	17
3	F	58	DM + RF	PDR + BRVO	B + C	LP	0.02	60	30	Fibrosis exudates in AC	12
4	F	62	DM + hypertension	CRVO	B + C, partial laser	HM	0.04	58	20	None	15
5	M	59	DM + RF	PDR + BRVO	B + C	CF	0.04	59	18	Temporary IOP elevation	14
6	F	61	DM + RF	PDR	B + C	0.08	0.1	48	17	None	17
7	M	63	DM + hypertension	CRVO	B + C	0.06	0.1	61	19	Fibrosis exudates in AC	12
8	M	63	DM + RF	PDR	B + C, partial laser	0.1	0.3	40	15	Fibrosis exudates in AC	13
9	F	59	DM + RF	PDR + BRVO	B + C	0.08	0.08	39	16	None	12
10	M	58	DM + hypertension	CRVO	B + C, partial laser	0.04	0.06	56	21	Fibrosis exudates in AC	12
11	F	60	DM + hypertension	PDR + BRVO	B + C	HM	0.02	58	19	SCH	24
12	F	63	DM + hypertension	PDR + BRVO	B + C	HM	0.08	57	18	None	13
13	M	59	DM + RF	PDR	B + C, partial laser	0.02	0.02	56	20	Fibrosis exudates in AC	17
14	M	71	Hypertension	CRVO	B + C	0.02	0.04	54	18	Fibrosis exudates in AC	12
15	F	63	DM + RF	PDR + BRVO	B + C	LP	0.04	64	19	Temporary IOP elevation	16
16	M	58	DM + hypertension	CRVO	B + C	CF	0.06	55	17	None	14
17	M	63	DM + RF	PDR	B + C	0.04	0.04	54	18	None	13
18	F	65	Hypertension	CRVO	B + C, partial laser	0.2	0.4	38	10	None	13

F = female; M = male; DM = diabetes mellitus; RF = renal failure; BCVA = best corrected visual acuity; AC = anterior chamber; SCH = suprachoroidal hemorrhage; B = brinzolamide; C = 2% carteolol hydrochloride.
